# Inflammation and the neural diathesis-stress hypothesis of schizophrenia: a reconceptualization

**DOI:** 10.1038/tp.2016.278

**Published:** 2017-02-07

**Authors:** O D Howes, R McCutcheon

**Affiliations:** 1 Department of Psychosis Studies, Institute of Psychiatry, Psychology & Neuroscience, King's College London, London, UK; 2 MRC London Institute of Medical Sciences, Hammersmith Hospital, London, UK; 3 Institute of Clinical Sciences, Faculty of Medicine, Imperial College London, London, UK

## Abstract

An interaction between external stressors and intrinsic vulnerability is one of the longest standing pathoaetiological explanations for schizophrenia. However, novel lines of evidence from genetics, preclinical studies, epidemiology and imaging have shed new light on the mechanisms that may underlie this, implicating microglia as a key potential mediator. Microglia are the primary immune cells of the central nervous system. They have a central role in the inflammatory response, and are also involved in synaptic pruning and neuronal remodeling. In addition to immune and traumatic stimuli, microglial activation occurs in response to psychosocial stress. Activation of microglia perinatally may make them vulnerable to subsequent overactivation by stressors experienced in later life. Recent advances in genetics have shown that variations in the complement system are associated with schizophrenia, and this system has been shown to regulate microglial synaptic pruning. This suggests a mechanism via which genetic and environmental influences may act synergistically and lead to pathological microglial activation. Microglial overactivation may lead to excessive synaptic pruning and loss of cortical gray matter. Microglial mediated damage to stress-sensitive regions such as the prefrontal cortex and hippocampus may lead directly to cognitive and negative symptoms, and account for a number of the structural brain changes associated with the disorder. Loss of cortical control may also lead to disinhibition of subcortical dopamine—thereby leading to positive psychotic symptoms. We review the preclinical and *in vivo* evidence for this model and consider the implications this has for treatment, and future directions.

## Introduction

A relationship between the social environment and mental illness has been recognized throughout the history of medicine, from Hippocrates through to the nineteenth century writings of Philippe Pinel and more recent literature.^1, 2^ The specific idea that a preexisting vulnerability and external stressors may interact in the pathogenesis of schizophrenia—the ‘diathesis-stress hypothesis'—was suggested over half a century ago.^3^ Subsequent refinements have attempted to define how this interaction might occur at a neurobiological level.

Walker and Diforio^4^ posited the hippocampus and hypothalamic-pituitary-adrenal (HPA) axis as the mediating pathway between environmental stressors, underlying vulnerability and development of the disorder. Specifically, pre- or perinatal neurodevelopmental insults were suggested to cause aberrant hippocampal function, while psychosocial stress exposure was posited to activate the HPA axis. Furthermore, dysregulation of the hippocampus and HPA axis were hypothesized to act synergistically, and activation of the HPA axis was asserted to stimulate the subcortical dopamine system, leading to the development of psychotic symptoms.

Van Winkel *et al.*^5^ extended this model by examining genetic factors underlying the proposed diathesis. Their review highlighted epidemiological studies that have shown a synergism between urbanicity and familial liability for psychosis,^6^ and between genetic risk and dysfunctional upbringing.^7^

In this paper, we review findings from the latest neuroimaging, genetic and preclinical work to provide an update of what has proven to be one of the longest standing pathoaetiological models for schizophrenia. In particular, we highlight how the immune system, and especially microglial cells, may have a central role.

## The immune system and glia

The three primary categories of glial cell are astrocytes, oligodendrocytes and microglia. Astrocytes ensure that the local cellular environment is appropriate for neuronal signaling, whereas oligodendrocytes are involved in the myelination of axons. Although the focus of the current review is on microglia, all three types of glial cell have been suggested as potentially having a pathoetiological role in schizophrenia.^8^

The role of the immune system in the pathoaetiology of mental illness has become increasingly recognized.^9, 10^ As well as the possibility of intrinsic immune abnormalities contributing to illness, the system is also a key pathway via which environmental factors influence central nervous system functioning. Microglia are the primary immune cells of the central nervous system. Quiescent microglial cells have multiple, motile, branch-like protrusions, that continually scan their local environment.^11^ Activation of microglia by environmental triggers leads to retraction of these protrusions, and enlargement of the cell body. Animal models show this occurs in response to immunological and traumatic stimuli, and also in response to psychosocial stress.^12, 13^ When activated they may release pro-inflammatory cytokines, or conversely have a role in suppressing inflammation.^14^

### The effects of activated microglia

**Figure 1:**
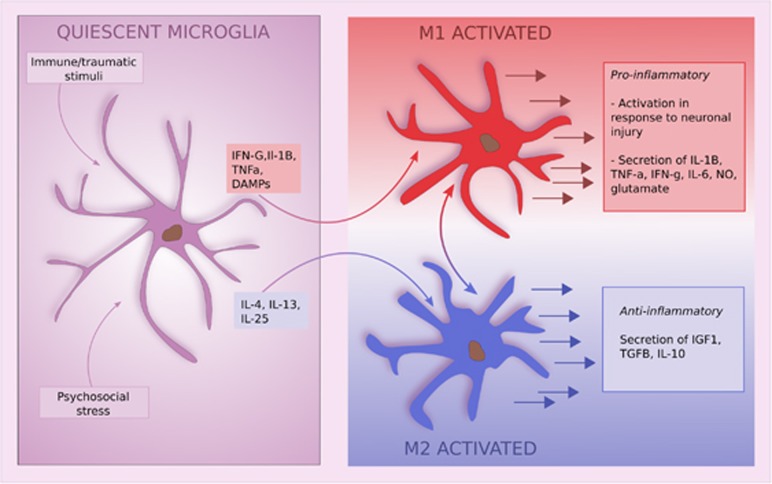
Activation of microglia and their subsequent effects. IL, interleukin; TNF, tumor necrosis factor.

Activated microglia exist on a continuum between two states, characterized as M1 and M2 activation, each with different molecular triggers.^15^ For example, M1 activation is triggered by cytokines such as IFN-G, interleukin (IL)1B, tumor necrosis factor (TNF)-a and damage-associated molecular patterns, whereas M2 activation is induced by cytokines such as IL-4, IL-13 and IL-25 (Figure 1).

The M1 pathway is activated following neuronal injury and leads to the release of a range of pro-inflammatory compounds including NO, IL-1B, TNF-a, IL-6 and glutamate. In a healthy system this is followed by a shift to the M2 state. This is broadly an anti-inflammatory pathway leading to release of IL-10, IGF-1, TGF-B, and various neurotrophic factors (Figure 1). The M2 pathway is involved in debris clearance, extracellular matrix deposition and angiogenesis.^14^ Both pathways are required for an appropriate immune response, and the balance between the two is tightly regulated in the healthy system.^14^

Dominance of the M1 pathway, with a prolonged inflammatory response, leads to over-expression of pro-inflammatory cytokines and reactive oxygen species, and thereby to synaptic loss and neuronal death.^16^ The possibility that the microglial response might be a cause, rather than solely a consequence of neuronal injury, was first suggested in Alzheimer's disease.^17^ Here a self-perpetuating mechanism was discovered whereby neuronal degeneration activates microglia, which then release neurotoxic molecules that cause further neuronal damage.^18^ Recently, prenatal immune activation was shown to be associated with a shift towards the M1 pathway in adolescence, and subsequent adult sensory gating deficits.^19^

It is important to note that the M1/M2 dichotomy is likely an over simplification of the various microglial states. Recent research has demonstrated the existence of dark microglia, a phenotype that is rarely seen under normal conditions, but is upregulated under chronic stress and may have a significant role in pathological pruning.^20^

### The role of microglia in cortical development and pruning

It has recently become clear that the role of microglia extends well beyond the inflammatory response. They promote survival of cortical neurons early in development via IGF-1 secretion,^21^ although conversely also demonstrate the ability to phagocytose neural precursor cells.^22^ As a result they are vital in regulating the pace and extent of neurogenesis in the developing brain.^23^ Moreover, they also have a role in synaptic pruning. This was first observed over 50 years ago,^24^ but recently it has become apparent that this is more extensive than originally thought. Microglial cells undertake constant synaptic monitoring; and rodent studies have demonstrated that pathological, and physiological pruning occurs throughout neurodevelopment and adult life.^25, 26^ There appears to be a fine balance between excessive and insufficient activity in this regard. Pathological reductions in microglial activity during neurodevelopment lead to reduced synaptic pruning, and sustained deficits in synaptic connectivity.^26, 27^ Conversely, microglial over-activity later in life has been linked to excessive synaptic loss and cognitive decline, and inhibition of microglial activity in this instance reduces the extent of pathological synaptic loss.^28^

### The effects of stress on microglia

Microglia are affected by a variety of stressors. In particular, ionized calcium binding adaptor molecule 1 (IBA-1) expression, a specific marker of microglial density, is increased in response to a number of stressors, including footshock, restraint, social defeat, maternal separation and social isolation.^29, 30^ This effect is seen in regions implicated in schizophrenia, including the amygdala, hippocampus, nucleus accumbens and prefrontal cortex. Interestingly, it appears that social defeat has the most marked impact upon IBA-1 expression.^29^

The role of glucocorticoids in the stress response is well established. Glucocorticoids (GCs) affect almost every immune cell type, due to the ubiquitous expression of the glucocorticoid receptor (GR). Within the central nervous system, microglia are a primary target for GCs due to their high level of GR expression.^31^ Research involving genetic manipulation of GR expression,^32^ and the administration of both GCs^33^ and GR antagonists,^34^ has demonstrated that GR signaling has a vital role in limiting the duration and amplitude of the microglial response. Paradoxically, animal models of acute and chronic stress (prior to an immune insult), have described a pro-inflammatory action of GCs.^35, 36^ Administration of a GR antagonist, or adrenalectomy has been shown to prevent the pro-inflammatory priming effects of stress on microglia.^35, 36^ GR activation of microglia also seems to be necessary for the expression of pro-inflammatory genes including IL-1B.^37^

A key factor in determining whether GCs have a pro- or anti-inflammatory effect is the timing relative to the inflammatory challenge (typically lipopolysaccharide (LPS) in experimental challenges). Administration of GCs prior to LPS has pro-inflammatory effects, whereas GC administration subsequent to LPS has anti-inflammatory effects.^38, 39, 40^ Interestingly, stress exposure subsequent to LPS administration appears to have anti-inflammatory effects as well.^38^

GC stimulation of neurons has been shown to increase glutamate release, one of the mechanisms potentially underlying stress induced cortical atrophy.^41^ This stress induced glutamate release has also been shown to result in microglial proliferation via activation of *N*-methyl-d-aspartate receptors (NMDAR).^42^ In a similar manner to the vicious circle described above in the case of Alzheimer's disease, microglial activation leads to neuronal damage, which causes further glutamate release and ongoing microglial activation.^43^

### Microglia and the perinatal period

Prenatal infection,^44^ neonatal infection,^45^ maternal stress^46^ and perinatal brain injury^47^ activate microglial and increase microglial densities in animal models (Box 1). As discussed below, microglia can have both a protective role (for example, their depletion has been shown to worsen post-hypoxia outcomes^48^), or contribute to pathology. Rats that experience neonatal infection show a blunted corticosterone response to stress in adulthood,^49^ which parallels the findings in individuals exposed to childhood trauma,^50^ and those with schizophrenia.^51, 52^

### Priming of microglia

‘Priming' refers to an exaggerated response to repeated presentations of a stimulus, compared with the initial response to the stimulus. This phenomenon has been observed repeatedly in microglia. Pre and postnatal stress, maternal immune activation, and neonatal infection lead to increased microglial activation and density.^46, 53, 54^ These changes later normalize.^55^ However, when subsequently exposed to an inflammatory stimulus in adult life, rats previously exposed to a perinatal hazard show an exaggerated microglial response.

Cross-sensitization of the microglial response has been shown between various stimuli.^46, 56^ Giovanoli *et al.*^57^ demonstrated a synergism in microglial response between perinatal insults, and adolescent stress. Maternal infection with a viral mimic was followed by five sequential peripubertal stressors. The group exposed to prenatal infection showed a threefold increase in markers of activated microglia in hippocampal and prefrontal areas in response to the peripubertal stress. This was secondary to reduced CD200 expression in the animals that had previously received a prenatal immune challenge (CD200 has a role in attenuating the inflammatory response, and is also downregulated following stress exposure^58^). The microglial response was not significantly different between any group when the stress exposure occurred in adulthood rather than the peripubertal period, suggesting there may be a critical developmental period outside of which the priming response does not occur.

### Critical developmental periods

Neuronal remodeling leading to an overall decrease in synaptic spine density is mediated by various mechanisms, including microglial pruning.^26^ In rodent studies the neonatal period is a period of peak microglia mediated pruning,^27^ although microglia have a role in this throughout the lifecourse.^59^ Humans may be unique, even among primates, in having a relatively late period of extensive synaptic remodeling during adolescence, that continues into adulthood.^60, 61, 62, 63^ Although rodent studies show microglia have a key role in synaptic pruning, this remains to be established in humans.

The neurodevelopmental time point at which exposure to a hazard occurs may significantly moderate the effect of that exposure. Bilbo *et al.*^54^ showed that neonatal infection at postnatal day (PND) 4 led to increased sensitivity to LPS exposure, but that this did not arise if infection occurred on PND 40. Other work examining later developments of seizures has also highlighted the early postnatal period as a time of particular vulnerability.^64^ It has also been demonstrated that changes in microglial density following in utero immune activation become evident in a window corresponding to adolescence, but may not be apparent at earlier or later timepoints.^65^ Moreover the Gionovali *et al.* study discussed above found that a primed response following perinatal immune activation only occurred if the stressor was delivered during adolesence.^57^ This indicates that in addition to being a period of extensive neuronal remodeling, adolescence represents a critical period for microglia that are already primed by prior activation to show an increased response to stress.^66^

## Schizophrenia and the environment

### Chronic and acute stress as a risk factor for schizophrenia

Epidemiological research has demonstrated associations between a wide range of psychosocial factors and schizophrenia. A history of 1st or 2nd generation migration, childhood trauma, and urbanicity have all been associated with schizophrenia with odds ratios of 2–4.^67^ For some environmental factors, such as obstetric complications, which may cause neuronal and white matter loss,^68^ a relatively direct neurobiological link between exposure and illness may exist. For others, however, such as urbanicity, migration and childhood adversity, the specific component of the exposure is harder to isolate. Nevertheless, studies have shown that social stressors significantly mediate the risk of psychosis associated with migration and urbanicity.^69, 70^ What these latter factors therefore have in common is that all involve exposure to chronic psychosocial stress.^71^

The role of acute stress in psychosis onset is well recognised clinically, and its role as a potential etiological factor in *acute and* transient psychotic disorder is described in the ICD-10 definition of the syndrome.^72^ However, an increase in the number of stressful events prior to psychosis onset has not been consistently demonstrated.^73^ This does not, however, rule out a role for acute stress, as the diathesis-stress model proposes that there is increased vulnerability to stress. Thus, there may be no difference in the acute stress exposure, but in vulnerable individuals this may trigger illness.^74^ This is supported by work demonstrating an increased incidence of psychosis associated with bombing campaigns during the 1999 Kosovo war,^75^ and more recently in refugees compared with non-refugee migrants.^76^ These studies have the advantage of investigating a relatively objective exposure, which addresses to some degree the possibility of reverse causality. In addition, in those with an established disorder, longitudinal studies have shown that there is an increase in the frequency of stressful life events prior to psychotic relapses.^77^

### Perinatal factors and infection

Prenatal infection,^78^ maternal inflammation during pregnancy,^79^ obstetric complications^80^ and childhood infections^81^ have all been associated with an increased risk of schizophrenia (Box 1). More recently a weak association between maternal stress during pregnancy and schizophrenia has been demonstrated,^82^ and it appears males may be particularly vulnerable.^83^ Recent epidemiological research has demonstrated a synergistic effect between prenatal infection and adolescent stress, in increasing schizophrenia risk, with the effect also predominantly in males.^84^ The parallels with the microglial findings discussed above are clearly apparent, and also of relevance is the influence of gender on microglial function—with male rats being particularly vulnerable to early-life infection-mediated microglial priming.^85^

Retrospective studies suggest that there is also an increased incidence of infection in adolescence and adulthood in individuals with schizophrenia.^86^ A prospective study in a military population demonstrated an association between antibodies to Toxoplasma Gondii evident in blood samples and later schizophrenia.^87^ Notwithstanding this, a paucity of longitudinal studies investigating infection prior to onset of schizophrenia, makes inferring the direction of causality a challenge.

## Schizophrenia and microglia

### Post mortem and *in vivo* imaging studies of microglia

Post-mortem studies in schizophrenia have used a variety of techniques to identify and characterize microglia.^88^ IBA-1, the marker elevated by stress exposure in the animal studies discussed above, has been used in two post mortem studies,^89, 90^ and these showed no difference in density of IBA-1 stained cells, although qualitative assessment of morphology found multiple activated microglia in schizophrenia samples that were not seen in control samples.^89^ Studies using other markers have shown increased microglia density, activation, and degeneration compared to controls^89, 91, 92, 93, 94, 95, 96^ (with some exceptions;^97, 98^ Supplementary Table 1). There is also evidence from two studies that microglial alterations are linked to the phenotype, with elevations seen in patients with paranoid symptoms but not in patients solely experiencing residual symptoms, suggesting microglial activation may be linked to active phases of the disorder.^99, 100^

*In vivo* imaging of microglia has used radioligands that bind to the translocator protein (TSPO), which is expressed on microglia and upregulated when they are activated. TSPO is, however, also expressed by cells other than microglia, such as endothelial cells and astrocytes,^101, 102, 103^ limiting both its sensitivity and specificity as a marker of microglial activation.

Table 1 summarizes the studies using this approach to index microglia in schizophrenia. The earliest two studies showed increased binding potentials in whole brain gray matter,^104^ and hippocampus,^105^ in individuals with schizophrenia. Later studies, however, have not consistently demonstrated an increase in binding.^106, 107, 108, 109, 110^ Meta-analysis has shown that there is a moderat effect size elevation in schizophrenia when binding potential is used as the outcome, but no effect when volume of distribution is used (Reis-Marques *et al.*, in submission). Methodological differences may account for this inconsistency between outcome measures.^111^ There is also preclinical evidence that antipsychotics may dampen microglia activity, raising the possibility that this could mask group differences in the studies of treated patients.^112, 113^ However, one preclinical study has found evidence antipsychotics increase microglial activity.^114^ An issue for the preclinical studies is that the dosing of antipsychotics does not reflect that used in patients, which limits translation. Further work using doses and modes of administration that reflect those used in patients is thus needed to determine the potential influence of antipsychotics for microglial activity in patients. Nevertheless, the only study to date in individuals at ultra-high risk for psychosis, who were all antipsychotic naïve, found increased relative binding in total gray matter, and in frontal and temporal regions.^115^

A number of studies have found associations between the magnitude of ligand binding and symptom severity. In ultra-high risk individuals, relative binding was directly correlated with symptom severity, and highest in the subject who subsequently developed a psychotic illness.^115^ Takano *et al.*^106^ found greater cortical binding potential was directly correlated with higher symptoms scores in schizophrenia, and Holmes *et al.* found that in a frontal cortical region it directly correlated with the PANSS-negative subscale, whereas, potentially paradoxically, Hafizi *et al.*^110^ found greater hippocampal binding correlated with better cognitive function. Although these findings suggest a link to symptoms, caution is warranted as not all correlations were corrected for multiple comparisons so there is a risk of false positives.

### Peripheral markers of inflammation

As described above microglial activation can have pro- or anti-inflammatory effects. Determining which pathway predominates in psychotic disorders *in vivo* is currently not possible as the available radioligands do not distinguish between M1 and M2 states.^117^ Nevertheless, evidence that there may be an imbalance in favor of the M1 pathway comes from studies examining peripheral cytokine levels.^118^ This suggests that medication-naive first-episode psychosis patients have increased expression of the M1 associated pro-inflammatory cytokines: IL-1B, IL-6 and TNFa.^119, 120^ Moreover, one of the triggers of M1 activation, S100B, is present at higher levels in individuals with schizophrenia.^121^ A parallel is seen here with childhood trauma in which raised levels of pro-inflammatory IL-6 and TNF-a,^122^ and reductions in brain-derived neurotrophic factor expression (a product of the M2 pathway) have been observed.^118, 120^

There is also evidence that alterations in inflammatory markers may exist well before the onset of psychosis, and may predict progression to psychosis.^123^ Post-mortem and neuroimaging studies in individuals with schizophrenia provide support for a link between immune activation and damage to both gray and white matter.^94, 118, 124, 125, 126^ In individuals with schizophrenia, an increase in peripheral cytokines associated with the M1 pathway has been shown to correlate with reductions in both hippocampal,^118^ and prefrontal cortex volumes.^124, 126^ A link between cytokine levels and TSPO binding, however, has not been demonstrated,^108^ which could be because cytokine levels fluctuate.

### Genetic findings

The largest genome-wide genetic association study (GWAS) to date identified multiple loci linked to the immune system among the strongest associations in the over 100 loci associated with schizophrenia.^127^ Although the potential impact of many of these has yet to be determined, one locus identified implicated the complement component 4 (C4).^128^ Alleles of this gene were subsequently shown by Sekar *et al.*^128^ to associate with schizophrenia in proportion to the amount of C4A that they generate, and greater expression of C4 in brains of individuals with schizophrenia was related to genotype. C4 activates complement component 3 (C3), allowing it to attach to a synapse. This marks the synapse for phagocytosis,^129^ and the complement receptor 3 (CR3) drives synaptic pruning by microglia.^27^ Sekar *et al.* went on to find that mice with the C4 alleles associated with greater C4 production showed elevated synaptic pruning during neurodevelopment.^128^ They also demonstrated that the C4 allele linked to schizophrenia determined the extent of C3 immunostaining thereby identifying an important genetic influence on the extent of microglial synaptic pruning.^128^ These results are an exciting development, and the first to provide a clear mechanistic pathway linked to the GWAS findings. However, for their full significance to be accepted, replication will be required.

## The potential role of microglia in an integrated model of the development of schizophrenia

Meta-analyses provide robust support for both dopamine dysfunction and reduced cortical gray matter in schizophrenia, including in medication-naive patients.^130, 131^ Although dysregulation of the dopaminergic system is thought to be central to the development of psychotic symptoms in schizophrenia,^67^ it is unclear what accounts for the loss of cortical synapses and cortical volume seen in schizophrenia. The lines of evidence we have reviewed suggest that microglia could explain this. First, in addition to affecting the development of dopaminergic neurons; perinatal insults, and early-life stress prime microglia to act in a hyper-responsive manner to later stress and encourage a shift to a pro-inflammatory M1 phenotype. Second, microglia have a significant role in pruning cortical synapses. Third, genetic variants in the complement pathway linked to schizophrenia have been shown to moderate microglial pruning.^128^ Thus in people with these genetic risk factors—subsequent stress, or immune activation could act on primed microglia, leading to overactivation and aberrant synaptic pruning.

**Figure 2:**
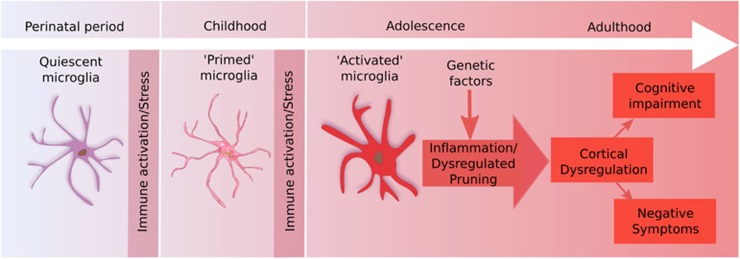
The ‘two hit' model. Perinatal activation of microglia leads to a primed state. Subsequent stress in adolescence triggers pathological overactivation, leading to cortical loss and the development of symptoms.

Microglial overactivation secondary to these ‘two hits' may then lead to spine loss via excessive pruning of stress-sensitive areas such as the prefrontal cortex and hippocampus (Figure 2). The loss of synapses due to this could account for the structural brain changes associated with schizophrenia and the development of negative and cognitive symptoms.^132^ This is supported by findings that lower gray matter volume is correlated with greater cognitive symptoms^133, 134^ and at least partially secondary to a reduction in the density of synapses.^135^

Furthermore, disrupted cortical development could exacerbate the disinhibition of subcortical dopamine neurons, which is thought to underlie the development of positive symptoms.^136, 137^ This would also have the effect of sensitizing the dopaminergic response to acute stress, creating a system unable to respond appropriately to acute stress, leading to further dysregulation. Interactions with genotype are also likely to occur at this point, for example, a polymorphism within the dopamine receptor 2 gene was also implicated in schizophrenia GWAS, and has been shown to moderate the dopaminergic response to stress.^138^

Although the model we present is wide ranging, we do not intend to suggest that microglia are the sole architects of the neurobiological abnormalities associated with schizophrenia and it is important to note the variability seen in the disorder. For example, although cognitive impairments and lower cortical gray matter volumes are consistent findings in schizophrenia, a proportion of patients show evidence of neither. Thus, it is likely that the schizophrenia syndrome encompasses several pathoaetiological pathways, which may co-occur in some but not all individuals. For example, stress^139^ and perinatal hazards^140^ may both directly act on the dopamine system to disinhibit it without involving microglia. This could lead to psychosis without marked cognitive impairments or gray matter reductions, although the involvement of the inflammatory system as well could account for the cortical volume loss, and negative and cognitive symptoms seen in other patients.

## Implications for treatment

Currently licensed treatments for schizophrenia all operate by blocking dopamine neurotransmission, and, while effective in controlling positive symptoms for some patients, they have little impact on cognitive or negative symptoms. There is a pressing need for novel treatment mechanisms, and in this regard microglia and the inflammatory response presents an attractive target, with the potential for modification of disease course, as opposed to solely symptomatic improvement.

A wide range of pharmacological agents have the ability to modify microglial function, including many existing psychotropics,^141, 142, 143^ and treatments originally developed for non-psychiatric indications such as statins, non-steroidal anti-inflammatories, N-acetyl cysteine, minocycline, and natalizumab.^144, 145, 146^ It is also possible that psychological interventions addressing stress reactivity could conceivably indirectly affect microglial function. There are a number of challenges, however, in developing interventions to modify microglial function. First, microglia have a vital physiological role, and attempts to inhibit their activity may potentially have deleterious effects.^26, 147^ Second, it appears that pathological overactivation of microglia occurs early in the course of the illness.^19, 57, 65^ Thus treatment may need to be given early, potentially during a prodromal period, to be effective and to prevent the secondary loss of synapses.

## Limitations and unanswered questions

A general issue for the field is that it is not clear how specific the findings discussed above are to psychotic disorders. The HPA axis, psychosocial stress, dopaminergic dysfunction, and microglial activation have been implicated in a wide range of mental illnesses. An interaction with genetic risk factors could explain different trajectories, and is supported by the findings linking genes in the complement pathway and the dopamine D2 receptor to schizophrenia. However, the interaction between these genetic and developmental risk factors, and alterations in microglial function has yet to be tested.

Moreover, although there is an extensive animal literature showing the impact of stress on microglia, research methods to investigate whether this corresponds to findings in humans are only starting to be developed, and remain limited by the specificity and resolution of the techniques available. In addition, strong evidence linking stress and microglial activity to negative and cognitive symptoms is lacking. Although we hypothesize that excessive pruning of cortical gray matter could lead to these symptoms, this requires testing. Although the finding that elevation in pro-inflammatory cytokines is associated with to gray matter loss provides some support for a link,^124^ a longitudinal multimodal approach will be required to determine whether microglia are causally implicated in the gray matter changes observed in schizophrenia.

In the current review, we have focused upon stress and infection as risk factors for schizophrenia. A number of other factors, however, have relevance both in terms of their relationship with microglia functioning, and the pathoaetiology of schizophrenia. Estradiol is thought to contribute to the gender differences in schizophrenia incidence, and is known to have anti-inflammatory effects.^148, 149^ Cannabis, meanwhile, has become increasingly accepted as having a causal role in increasing schizophrenia risk, and has been shown to activate microglia.^150, 151^ In addition, a wide range of non-dopamine neurotransmitter systems may be involved in the development of psychosis.^152, 153^ Box 2 highlights future directions to address the issues discussed above.

Current human imaging and post-mortem studies of microglia show inconsistency. Several reasons may underlie this. First, schizophrenia is a heterogeneous concept that likely encompasses various aetiologies, this is highlighted by the recent finding that peripheral markers of inflammation show marked differences between responders and non-responders to antipsychotic treatment.^154^ In addition to this inter-individual variability, intra-individual temporal variability is suggested by the findings of Giovanoli *et al.* that microglial changes may only be present at specific time points (for example, during adolescence).^57^

## Conclusions

The importance of environmental stressors in the development of schizophrenia has been recognized for longer than our current classifications of mental illness. Over recent years, studies have shown the impact of these risk factors on the immune system. In the present review we draw on these lines of evidence to suggest how microglial cells in particular may have a role in the pathoaetiology of schizophrenia.

Evidence shows that microglial cells may become primed early in life, making them vulnerable to subsequent chronic overactivation following further stimulation. This may then cause gray matter loss in regions such as the prefrontal cortex and hippocampus, leading to negative and cognitive symptoms, and potentially contributing to the dopaminergic dysregulation of subcortical structures. The wealth of evidence supporting the link between perinatal and later life risk factors and schizophrenia, the elevation in pro-inflammatory cytokines including those associated with M1-activated microglia in schizophrenia, and the elevation in microglia seen with stress mean we can be fairly confident about these aspects of the model. Nevertheless, it is important to recognize that the role of microglia in the disorder, and their link to other elements of its pathology, requires further testing.

Although we have concentrated on psychotic disorders, it is clear that many of the mechanisms described above do not segregate according to traditional diagnostic boundaries. The mechanisms we describe present a wealth of targets for potential therapeutic intervention, for many mental illnesses. However, their complexity and wide ranging effects means producing targeted interventions will be a significant challenge.

## Figures and Tables

**Table 1:** Imaging studies of translocator protein density in individuals with psychotic disorders

*Study*	*Population*	*Patient age, mean (s.d.)*	*Medication*	*Methods*	*Findings*
Van Berckel *et al.* (2008)^104^	10 Scz within 5 years of disease onset. 10 HC	24 (2)	All patients antipsychotic treated	*Ligand*: (R)-[11C]PK11195 *TSPO genotype*: not measured	Significantly greater whole brain gray matter BP in patients (*d*=0.87)
Doorduin *et al.* (2009)^105^	7 Scz. (Mean PANSS 74) 8 HC	31 (7)	All patients antipsychotic treated	*Ligand:* (R)-[11C]PK11195 *TSPO genotype*: not measured	Hippocampal BP significantly greater in patients (*d*=1.92) Whole brain gray matter non-significantly greater (*d*= 0.84)
Takano *et al.* (2010)^106^	14 Scz (Mean PANSS 78.6) 14 HC	43.9 (7.4)	All patients antipsychotic treated	*Ligand*: [11C]DAA1106 *TSPO genotype*: not measured	No significant differences in BP_ND_ between groups. BP_ND_ directly correlated with symptoms score.
Bloomfield *et al.* (2015)^115^	14 UHR (Mean CAARMS 49.5) 14 HC	24	No antipsychotic exposure	*Ligand*: [11C]PBR28 *TSPO genotype*: controlled for	Vtr elevated for UHR for total GM (*d*=1.2), frontal lobe (*d*=0.89) and temporal lobe (*d*=0.83). No difference between groups in Vt.
	14 Scz (Mean PANSS 63.7) 14 HC	47	Antipsychotic treated		Vtr elevated for Scz for total GM (*d*=1.77), frontal lobe (*d*=1.25) and temporal lobe (*d*=1.43). No difference between groups in terms of Vt.
Kenk *et al.* (2015)^107^	16 Scz (Mean PANSS 70.2) 27 HC	43 (14.0)	All patients antipsychotic treated	*Ligand*: [18F]-FEPPA *TSPO genotype*: controlled for	No significant differences in whole brain or ROIS white or gray matter Vt.
Coughlin *et al.* (2016)^108^	12 Scz (Mean SAPS 3.8) 14 HC	24.1 (3.1)	All patients antipsychotic treated.	*Ligand*: [11C]DPA-713 *TSPO genotype*: controlled for	No significant differences in whole brain or ROIS white or gray matter Vt.
van der Doef *et al.* (2016)^109^	19 Psychotic disorder (Mean PANSS 53) 17 HC	26 (4)	15/19 antipsychotic treated	*Ligand*: (R)-[11C]PK11195 *TSPO genotype*: not measured	No significant differences in whole brain or ROIS BP_ND_
Hafizi *et al.* (2016)^110^	19 FEP (Mean PANSS 68.6) 20 HC	27.5 (6.7)	All <4 weeks lifetime antipsychotic exposure and 14 antipsychotic naïve	*Ligand*: [18 F]-FEPPA *TSPO genotype*: controlled for	No significant differences in whole brain or ROIS Vt.
Holmes *et al.* (2016)^116^	16 Scz 16 HC	32.5	8 antipsychotic free 8 antipsychotic treated	*Ligand*: (R)-[11C]PK11195 *TSPO genotype*: not measured	Cortical BP_ND_ significantly higher in medicated patients than in controls. No difference between unmedicated patients and controls.

Abbreviations: BP, binding potential; CAARMS, Comprehensive Assessment of the At-Risk Mental States; FEP, first-episode psychosis; HC, healthy control; PANSS, Positive and Negative Syndrome Scale; SAPS, Scale for the Assessment of Positive Symptoms; UHR, ultra-high risk; Vt, volume of distribution; Vtr, ratio of Vt in the region of interest to the Vt of whole brain.

## Directions for future research

Preclinical research

- Determine effects of antipsychotic on microglia using doses and modes of administration that appropriately reflect human use.
- Conduct studies using translational approaches (such as MR and PET imaging and peripheral blood measures) that can inform interpretation of human studies

General

- Develop and validate approaches to the analysis of current PET tracers for TSPO that address methodological issues
- Develop novel radioligands with greater specificity for microglia and that will distinguish between M1 and M2 states.

Clinical Research

- Multimodal longitudinal studies to determine the relationship between microglia and other brain changes as well as the effect of moderating factors (such as sex hormone levels)
- Comparative studies across disorders to determine common and specific mechanisms
- *In vivo* testing of how risk factors for psychosis (such as stress, cannabis use, migration) impact microglial function
- Determine the effects of antipsychotic treatment upon microglial activity in longitudinal studies
- Experimental medicine studies of the effects of modulating microglial function in schizophrenia on gray matter changes and symptoms

## References

[bib1] Pinel P. Traité médico-philosophique sur l'aliénation mentale, ou la manie. Richard Caille et Ravier: Paris,1801.

[bib2] Angst J, Marneros A. Bipolarity from ancient to modern times. J Affect Disord 2001; 67: 3–19. 11869749 10.1016/s0165-0327(01)00429-3

[bib3] Meehl PE. Schizotaxia, schizotypy, schizophrenia. Am Psychol 1962; 17: 827–838.

[bib4] Walker EF, Diforio D. Schizophrenia: a neural diathesis-stress model. Psychol Rev 1997; 104: 667–685. 9337628 10.1037/0033-295x.104.4.667

[bib5] Van Winkel R, Stefanis NC, Myin-Germeys I. Psychosocial stress and psychosis. A review of the neurobiological mechanisms and the evidence for gene-stress interaction. Schizophr Bull 2008; 34: 1095–1105. 18718885 10.1093/schbul/sbn101 PMC2632486

[bib6] Van Os J, Pedersen CB, Mortensen PB. Confirmation of synergy between urbanicity and familial liability in the causation of psychosis. Am J Psychiatry 2004; 161: 2312–2314. 15569906 10.1176/appi.ajp.161.12.2312

[bib7] Tienari P, Ynne LCW, Sorri A, Lahti I. Genotype -environment interaction in schizophrenia-spectrum disorder Long-term follow-up study of Finnish adoptees. Br J Psychiatry 2004; 184: 216–222. 14990519 10.1192/bjp.184.3.216

[bib8] Bernstein H-G, Steiner J, Bogerts B. Glial cells in schizophrenia: pathophysiological significance and possible consequences for therapy. Expert Rev Neurother 2009; 9: 1059–1071. 19589054 10.1586/ern.09.59

[bib9] Khandaker GM, Cousins L, Deakin J, Lennox BR, Yolken R, Jones PB. Inflammation and immunity in schizophrenia: Implications for pathophysiology and treatment. Lancet Psychiatry 2015; 2: 258–270. 26359903 10.1016/S2215-0366(14)00122-9 PMC4595998

[bib10] Rosenblat JD, Cha DS, Mansur RB, McIntyre RS. Inflamed moods: a review of the interactions between inflammation and mood disorders. Prog Neuro-Psychopharmacology Biol Psychiatry 2014; 53: 23–34. 10.1016/j.pnpbp.2014.01.013 24468642

[bib11] Nimmerjahn A, Kirchhoff F, Helmchen F. Resting microglial cells are highly dynamic surveillants of brain parenchyma *in vivo*—resting microglial cells are highly dynamic surveillants of brain parenchyma in v*ivo*. Science 2005; 308: 1314–1319. 15831717 10.1126/science.1110647

[bib12] Hinwood M, Morandini J, Day TA, Walker FR. Evidence that microglia mediate the neurobiological effects of chronic psychological stress on the medial prefrontal cortex. Cereb Cortex 2012; 22: 1442–1454. 21878486 10.1093/cercor/bhr229

[bib13] Tynan RJ, Naicker S, Hinwood M, Nalivaiko E, Buller KM, Pow D V et al. Chronic stress alters the density and morphology of microglia in a subset of stress-responsive brain regions. Brain Behav Immun 2010; 24: 1058–1068. 20153418 10.1016/j.bbi.2010.02.001

[bib14] Cherry JD, Olschowka JA, O'Banion MK. Neuroinflammation and M2 microglia: the good, the bad, and the inflamed. J Neuroinflammation 2014; 11: 98. 24889886 10.1186/1742-2094-11-98 PMC4060849

[bib15] Colton CA. Heterogeneity of microglial activation in the innate immune response in the brain. J Neuroimmune Pharmacol 2009; 4: 399–418. 19655259 10.1007/s11481-009-9164-4 PMC2773116

[bib16] Rao JS, Kellom M, Kim HW, Rapoport SI, Reese EA. Neuroinflammation and synaptic loss. Neurochem Res 2012; 37: 903–910. 22311128 10.1007/s11064-012-0708-2 PMC3478877

[bib17] Griffin WS, Stanley LC, Ling C, White L, MacLeod V, Perrot LJ et al. Brain interleukin 1 and S-100 immunoreactivity are elevated in Down syndrome and Alzheimer disease. Proc Natl Acad Sci USA 1989; 86: 7611–7615. 2529544 10.1073/pnas.86.19.7611 PMC298116

[bib18] Akiyama H, Barger S, Barnum S, Bradt B, Bauer J, Cole GM et al. Inflammation and Alzheimer's disease. Neurobiol Aging 2000; 21: 383–421. 10858586 10.1016/s0197-4580(00)00124-x PMC3887148

[bib19] Eßlinger M, Wachholz S, Manitz M-P, Plümper J, Sommer R, Juckel G et al. Schizophrenia associated sensory gating deficits develop after adolescent microglia activation. Brain Behav Immun 2016; 58: 99–106. 27235930 10.1016/j.bbi.2016.05.018

[bib20] Bisht K, Sharma KP, Lecours C, Gabriela Sánchez M, El Hajj H, Milior G et al. Dark microglia: a new phenotype predominantly associated with pathological states. Glia 2016; 64: 826–839. 26847266 10.1002/glia.22966 PMC4949554

[bib21] Ueno M, Fujita Y, Tanaka T, Nakamura Y, Kikuta J, Ishii M et al. Layer V cortical neurons require microglial support for survival during postnatal development. Nat Neurosci 2013; 16: 543–551. 23525041 10.1038/nn.3358

[bib22] Cunningham CL, Martinez-Cerdeno V, Noctor SC. Microglia regulate the number of neural precursor cells in the developing cerebral cortex. J Neurosci 2013; 33: 4216–4233. 23467340 10.1523/JNEUROSCI.3441-12.2013 PMC3711552

[bib23] Michell-Robinson MA, Touil H, Healy LM, Owen DR, Durafourt BA, Bar-Or A et al. Roles of microglia in brain development, tissue maintenance and repair. Brain 2015; 138: 1138–1159. 25823474 10.1093/brain/awv066 PMC5963417

[bib24] Blinzinger K, Kreutzberg G. Displacement of synaptic terminals from regenerating motoneurons by microglial cells. Zeitschrift fur Zellforsch und Mikroskopische Anat 1968; 85: 145–157. 10.1007/BF00325030 5706753

[bib25] Kettenmann H, Kirchhoff F, Verkhratsky A. Microglia: new roles for the synaptic stripper. Neuron 2013; 77: 10–18. 23312512 10.1016/j.neuron.2012.12.023

[bib26] Paolicelli RC, Bolasco G, Pagani F, Maggi L, Scianni M, Panzanelli P et al. Synaptic pruning by microglia is necessary for normal brain development. Science 2011; 333: 1456–1458. 21778362 10.1126/science.1202529

[bib27] Schafer DP, Lehrman EK, Kautzman AG, Koyama R, Mardinly AR, Yamasaki R et al. Microglia sculpt postnatal neural circuits in an activity and complement-dependent manner. Neuron 2012; 74: 691–705. 22632727 10.1016/j.neuron.2012.03.026 PMC3528177

[bib28] Hong S, Beja-Glasser VF, Nfonoyim BM, Frouin A, Li S, Ramakrishnan S et al. Complement and microglia mediate early synapse loss in Alzheimer mouse models. Science 2016; 352: 712–716. 27033548 10.1126/science.aad8373 PMC5094372

[bib29] Calcia MA, Bonsall DR, Bloomfield PS, Selvaraj S, Barichello T, Howes OD. Stress and neuroinflammation: a systematic review of the effects of stress on microglia and the implications for mental illness. Psychopharmacology (Berl) 2016; 233: 1637–1650. 26847047 10.1007/s00213-016-4218-9 PMC4828495

[bib30] Delpech J-C, Wei L, Hao J, Yu X, Madore C, Butovsky O et al. Early life stress perturbs the maturation of microglia in the developing hippocampus. Brain Behav Immun 2016; 57: 79–93. 27301858 10.1016/j.bbi.2016.06.006 PMC5010940

[bib31] Sierra A, Gottfried-Blackmore A, Milner TA, McEwen BS, Bulloch K. Steroid hormone receptor expression and function in microglia. Glia 2008; 56: 659–674. 18286612 10.1002/glia.20644

[bib32] Ros-Bernal F, Hunot S, Herrero MT, Parnadeau S, Corvol J-C, Lu L et al. Microglial glucocorticoid receptors play a pivotal role in regulating dopaminergic neurodegeneration in parkinsonism. Proc Natl Acad Sci USA 2011; 108: 6632–6637. 21467220 10.1073/pnas.1017820108 PMC3080980

[bib33] Drew PD, Chavis JA. Inhibition of microglial cell activation by cortisol. Brain Res Bull 2000; 52: 391–396. 10922518 10.1016/s0361-9230(00)00275-6

[bib34] Blais V, Turrin NP, Rivest S. Cyclooxygenase 2 (COX-2) inhibition increases the inflammatory response in the brain during systemic immune stimuli. J Neurochem 2005; 95: 1563–1574. 16277613 10.1111/j.1471-4159.2005.03480.x

[bib35] Frank MG, Thompson BM, Watkins LR, Maier SF. Glucocorticoids mediate stress-induced priming of microglial pro-inflammatory responses. Brain Behav Immun 2012; 26: 337–345. 22041296 10.1016/j.bbi.2011.10.005 PMC5652300

[bib36] Frank MG, Hershman SA, Weber MD, Watkins LR, Maier SF. Chronic exposure to exogenous glucocorticoids primes microglia to pro-inflammatory stimuli and induces NLRP3 mRNA in the hippocampus. Psychoneuroendocrinology 2014; 40: 191–200. 24485491 10.1016/j.psyneuen.2013.11.006 PMC3912460

[bib37] Glezer I, Simard AR, Rivest S. Neuroprotective role of the innate immune system by microglia. Neuroscience 2007; 147: 867–883. 17459594 10.1016/j.neuroscience.2007.02.055

[bib38] Goujon E, Parnet P, Laye S, Combe C, Kelley KW, Dantzer R. Stress downregulates lipopolysaccharide-induced expression of proinflammatory cytokines in the spleen, pituitary, and brain of mice. Brain Behav Immun 1995; 9: 292–303. 8903847 10.1006/brbi.1995.1028

[bib39] Frank MG, Watkins LR, Maier SF. The permissive role of glucocorticoids in neuroinflammatory priming. Curr Opin Endocrinol Diabetes Obes 2015; 22: 1. 26087336 10.1097/MED.0000000000000168 PMC4516217

[bib40] Frank MG, Weber MD, Watkins LR, Maier SF. Stress-induced neuroinflammatory priming: a liability factor in the etiology of psychiatric disorders. Neurobiol Stress 2016; 4: 62–70. 27981190 10.1016/j.ynstr.2015.12.004 PMC5146200

[bib41] Moghaddam B, Bolinao ML, Stein-Behrens B, Sapolsky R. Glucocortcoids mediate the stress-induced extracellular accumulation of glutamate. Brain Res 1994; 655: 251–254. 7812782 10.1016/0006-8993(94)91622-5

[bib42] Nair A, Bonneau RH. Stress-induced elevation of glucocorticoids increases microglia proliferation through NMDA receptor activation. J Neuroimmunol 2006; 171: 72–85. 16278020 10.1016/j.jneuroim.2005.09.012

[bib43] Kaindl AM, Degos V, Peineau S, Gouadon E, Chhor V, Loron G et al. Activation of microglial N-methyl-D-aspartate receptors triggers inflammation and neuronal cell death in the developing and mature brain. Ann Neurol 2012; 72: 536–549. 23109148 10.1002/ana.23626

[bib44] Boksa P. Effects of prenatal infection on brain development and behavior: a review of findings from animal models. Brain Behav Immun 2010; 24: 881–897. 20230889 10.1016/j.bbi.2010.03.005

[bib45] Sominsky L, Walker AK, Ong LK, Tynan RJ, Walker FR, Hodgson DM. Increased microglial activation in the rat brain following neonatal exposure to a bacterial mimetic. Behav Brain Res 2012; 226: 351–356. 21907243 10.1016/j.bbr.2011.08.038

[bib46] Diz-Chaves Y, Astiz M, Bellini MJ, Garcia-Segura LM. Prenatal stress increases the expression of proinflammatory cytokines and exacerbates the inflammatory response to LPS in the hippocampal formation of adult male mice. Brain Behav Immun 2013; 28: 196–206. 23207108 10.1016/j.bbi.2012.11.013

[bib47] Hagberg H, Mallard C, Ferriero DM, Vannucci SJ, Levison SW, Vexler ZS et al. The role of inflammation in perinatal brain injury. Nat Rev Neurol 2015; 11: 192–208. 25686754 10.1038/nrneurol.2015.13 PMC4664161

[bib48] Faustino J V, Wang X, Johnson CE, Klibanov A, Derugin N, Wendland MF et al. Microglial cells contribute to endogenous brain defenses after acute neonatal focal stroke. J Neurosci 2011; 31: 12992–13001. 21900578 10.1523/JNEUROSCI.2102-11.2011 PMC3539822

[bib49] Bilbo SD, Yirmiya R, Amat J, Paul ED, Watkins LR, Maier SF. Bacterial infection early in life protects against stressor-induced depressive-like symptoms in adult rats. Psychoneuroendocrinology 2008; 33: 261–269. 18164556 10.1016/j.psyneuen.2007.11.008 PMC2274778

[bib50] Danese A, McEwen BS. Adverse childhood experiences, allostasis, allostatic load, and age-related disease. Physiol Behav 2012; 106: 29–39. 21888923 10.1016/j.physbeh.2011.08.019

[bib51] Borges S, Gayer-Anderson C, Mondelli V. A systematic review of the activity of the hypothalamic-pituitary-adrenal axis in first episode psychosis. Psychoneuroendocrinology 2013; 38: 603–611. 23369532 10.1016/j.psyneuen.2012.12.025

[bib52] Aiello G, Horowitz M, Hepgul N, Pariante CM, Mondelli V. Stress abnormalities in individuals at risk for psychosis: A review of studies in subjects with familial risk or with ‘ at risk' mental state. Psychoneuroendocrinology 2012; 37: 1600–1613. 22663896 10.1016/j.psyneuen.2012.05.003

[bib53] Ling Z, Zhu Y, Tong CW, Snyder JA, Lipton JW, Carvey PM. Prenatal lipopolysaccharide does not accelerate progressive dopamine neuron loss in the rat as a result of normal aging. Exp Neurol 2009; 216: 312–320. 19133261 10.1016/j.expneurol.2008.12.004

[bib54] Bilbo SD. Neonatal Infection-Induced memory impairment after lipopolysaccharide in adulthood is prevented via caspase-1 inhibition. J Neurosci 2005; 25: 8000–8009. 16135757 10.1523/JNEUROSCI.1748-05.2005 PMC6725459

[bib55] Gómez-González B, Escobar A. Prenatal stress alters microglial development and distribution in postnatal rat brain. Acta Neuropathol 2010; 119: 303–315. 19756668 10.1007/s00401-009-0590-4

[bib56] Diz-Chaves Y, Pernía O, Carrero P, Garcia-Segura LM. Prenatal stress causes alterations in the morphology of microglia and the inflammatory response of the hippocampus of adult female mice. J Neuroinflamm 2012; 9: 71. 10.1186/1742-2094-9-71 PMC3409032 22520439

[bib57] Giovanoli S, Engler H, Engler A, Richetto J, Voget M, Willi R et al. Stress in puberty unmasks latent neuropathological consequences of prenatal immune activation in mice. Science 2013; 339: 1095–1099. 23449593 10.1126/science.1228261

[bib58] Frank MG, Baratta M V, Sprunger DB, Watkins LR, Maier SF. Microglia serve as a neuroimmune substrate for stress-induced potentiation of CNS pro-inflammatory cytokine responses. Brain Behav Immun 2007; 21: 47–59. 16647243 10.1016/j.bbi.2006.03.005 PMC13674783

[bib59] Tremblay M-E, Stevens B, Sierra A, Wake H, Bessis A, Nimmerjahn A. The role of microglia in the healthy brain. J Neurosci 2011; 31: 16064–16069. 22072657 10.1523/JNEUROSCI.4158-11.2011 PMC6633221

[bib60] Petanjek Z, Judas M, Simic G, Rasin MR, Uylings HBM, Rakic P et al. Extraordinary neoteny of synaptic spines in the human prefrontal cortex. Proc Natl Acad Sci USA 2011; 108: 13281–13286. 21788513 10.1073/pnas.1105108108 PMC3156171

[bib61] Huttenlocher PR. Synaptic density in human frontal cortex - developmental changes and effects of aging. Brain Res 1979; 163: 195–205. 427544 10.1016/0006-8993(79)90349-4

[bib62] Whitaker KJ, Vértes PE, Romero-Garcia R, Váša F, Moutoussis M, Prabhu G et al. Adolescence is associated with transcriptionally patterned consolidation of the hubs of the human brain connectome. Proc Natl Acad Sci USA 2016; 113: 9105–9110. 27457931 10.1073/pnas.1601745113 PMC4987797

[bib63] Harris LW, Lockstone HE, Khaitovich P, Weickert CS, Webster MJ, Bahn S. Gene expression in the prefrontal cortex during adolescence: implications for the onset of schizophrenia. BMC Med Genomics 2009; 2: 28. 19457239 10.1186/1755-8794-2-28 PMC2694209

[bib64] Harry GJ, Kraft AD. Microglia in the developing brain: a potential target with lifetime effects. Neurotoxicology 2012; 33: 191–206. 22322212 10.1016/j.neuro.2012.01.012 PMC3299893

[bib65] Manitz MP, Esslinger M, Wachholz S, Plümper J, Friebe A, Juckel G et al. The role of microglia during life span in neuropsychiatric disease - an animal study. Schizophr Res 2013; 143: 221–222. 23182726 10.1016/j.schres.2012.10.028

[bib66] Gogtay N, Vyas NS, Testa R, Wood SJ, Pantelis C. Age of onset of Schizophrenia: perspectives from structural neuroimaging studies. Schizophr Bull 2011; 37: 504–513. 21505117 10.1093/schbul/sbr030 PMC3080674

[bib67] Howes O, Murray R. Schizophrenia: an integrated sociodevelopmental-cognitive model. Lancet 2014; 6736: 1–11. 10.1016/S0140-6736(13)62036-X PMC4127444 24315522

[bib68] Froudist-Walsh S, Karolis V, Caldinelli C, Brittain PJ, Kroll J, Rodriguez-Toscano E et al. Very early brain damage leads to remodeling of the working memory system in adulthood: A Combined fMRI/Tractography Study. J Neurosci 2015; 35: 15787–15799. 26631462 10.1523/JNEUROSCI.4769-14.2015 PMC4666909

[bib69] Heinz A, Deserno L, Reininghaus U. Urbanicity, social adversity and psychosis. World Psychiatry 2013; 12: 187–197. 24096775 10.1002/wps.20056 PMC3799240

[bib70] Morgan C, Kirkbride J, Hutchinson G, Craig T, Morgan K, Dazzan P et al. Cumulative social disadvantage, ethnicity and first-episode psychosis: a case-control study. Psychol Med 2008; 38: 1701–1715. 19000327 10.1017/S0033291708004534

[bib71] Lederbogen F, Haddad L, Meyer-Lindenberg A. Urban social stress—risk factor for mental disorders. The case of schizophrenia. Environ Pollut 2013; 183: 2–6. 23791151 10.1016/j.envpol.2013.05.046

[bib72] World Health Organization. The ICD-10 classification of mental and behavioural disorders. Geneva,1992.

[bib73] Phillips LJ, Francey SM, Edwards J, McMurray N. Stress and psychosis: towards the development of new models of investigation. Clin Psychol Rev 2007; 27: 307–317. 17169470 10.1016/j.cpr.2006.10.003

[bib74] Norman RM, Malla AK. Stressful life events and schizophrenia. I: A review of the research. Br J Psychiatry 1993; 162: 161–166. 8435685 10.1192/bjp.162.2.161

[bib75] Zolkowska K, Cantor-Graae E, McNeil TF. Psychiatric admissions for psychosis in Malmö during the NATO bombing of Kosovo. J Nerv Ment Dis 2003; 191: 820–826. 14671459 10.1097/01.nmd.0000100926.46390.4c

[bib76] Hollander A-C, Dal H, Lewis G, Magnusson C, Kirkbride JB, Dalman C. Refugee migration and risk of schizophrenia and other non-affective psychoses: a cohort study of 1.3 m people in Sweden. BMJ Br Med J 2016; 352: i1030. 26979256 10.1136/bmj.i1030 PMC4793153

[bib77] Mondelli V. From stress to psychosis: whom, how, when and why? Epidemiol Psychiatr Sci 2014; 23: 215–218. 24905592 10.1017/S204579601400033X PMC6998263

[bib78] Brown AS, Derkits EJ. Prenatal infection and schizophrenia: A review of epidemiologic and translational studies. Am J Psychiatry 2010; 167: 261–280. 20123911 10.1176/appi.ajp.2009.09030361 PMC3652286

[bib79] Canetta S, Sourander A. Elevated maternal C-reactive protein and increased risk of schizophrenia in a national birth cohort. Am J Psychiatry 2014; 171: 960–968. 24969261 10.1176/appi.ajp.2014.13121579 PMC4159178

[bib80] Cannon M, Jones PB, Murray RM. Reviews and overviews obstetric complications and Schizophrenia: historical and meta-analytic review. Am J Psychiatry 2002; 159: 1080–1092. 12091183 10.1176/appi.ajp.159.7.1080

[bib81] Khandaker GM, Zimbron J, Dalman C, Lewis G, Jones PB. Childhood infection and adult schizophrenia: A meta-analysis of population-based studies. Schizophr Res 2012; 139: 161–168. 22704639 10.1016/j.schres.2012.05.023 PMC3485564

[bib82] Dorrington S, Zammit S, Asher L, Evans J, Heron J, Lewis G. Perinatal maternal life events and psychotic experiences in children at twelve years in a birth cohort study. Schizophr Res 2014; 152: 158–163. 24275580 10.1016/j.schres.2013.11.006 PMC3906533

[bib83] Fineberg AM, Ellman LM, Schaefer CA, Maxwell SD, Shen L, H. Chaudhury N et al. Fetal exposure to maternal stress and risk for schizophrenia spectrum disorders among offspring: differential influences of fetal sex. Psychiatry Res 2015; 236: 91–97. 26753951 10.1016/j.psychres.2015.12.026 PMC4767153

[bib84] Debost J-CPG, Larsen JT, Munk-Olsen T, Mortensen PB, Meyer U, Petersen L. Joint effects of exposure to prenatal infection and peripubertal psychological trauma in Schizophrenia. Schizophr Bull 2017 (in press). 10.1093/schbul/sbw083 PMC5216853 27343007

[bib85] Bilbo SD, Smith SH, Schwarz JM. A lifespan approach to neuroinflammatory and cognitive disorders: A critical role for glia. J Neuroimmune Pharmacol 2012; 7: 24–41. 21822589 10.1007/s11481-011-9299-y PMC3267003

[bib86] Brown AS. The environment and susceptibility to schizophrenia. Prog Neurobiol 2011; 93: 23–58. 20955757 10.1016/j.pneurobio.2010.09.003 PMC3521525

[bib87] Niebuhr DW, Millikan AM, Cowan DN, Yolken R, Li Y. Selected infectious agents and risk of Schizophrenia among U. S. Military Personnel. Am J Psychiatry 2008; 165: 99–106. 18086751 10.1176/appi.ajp.2007.06081254

[bib88] Trépanier MO, Hopperton KE, Mizrahi R, Mechawar N, Bazinet RP. Postmortem evidence of cerebral inflammation in schizophrenia: a systematic review. Mol Psychiatry 2016; 21: 1009–1026. 27271499 10.1038/mp.2016.90 PMC4960446

[bib89] Hercher C, Chopra V, Beasley CL. Evidence for morphological alterations in prefrontal white matter glia in schizophrenia and bipolar disorder. J Psychiatry Neurosci 2014; 39: 376–385. 24936776 10.1503/jpn.130277 PMC4214872

[bib90] Connor CM, Guo Y, Akbarian S. Cingulate white matter neurons in schizophrenia and bipolar disorder. Biol Psychiatry 2009; 66: 486–493. 19559403 10.1016/j.biopsych.2009.04.032 PMC2725195

[bib91] Wierzba-Bobrowicz T, Lewandowska E, Lechowicz W, Stepień T, Pasennik E. Quantitative analysis of activated microglia, ramified and damage of processes in the frontal and temporal lobes of chronic schizophrenics. Folia Neuropathol 2005; 43: 81–89. 16012909

[bib92] Wierzba-Bobrowicz T, Lewandowska E, Kosno-Kruszewska E, Lechowicz W, Pasennik E, Schmidt-Sidor B. Degeneration of microglial cells in frontal and temporal lobes of chronic schizophrenics. Folia Neuropathol 2004; 42: 157–165. 15535034

[bib93] Uranova Na, Vikhreva OV, Rachmanova VI, Orlovskaya DD. Ultrastructural alterations of myelinated fibers and oligodendrocytes in the prefrontal cortex in Schizophrenia: a Postmortem Morphometric Study. Schizophr Res Treatment 2011; 2011: 1–13. 10.1155/2011/325789 PMC3420756 22937264

[bib94] Fillman SG, Cloonan N, Catts VS, Miller LC, Wong J, McCrossin T et al. Increased inflammatory markers identified in the dorsolateral prefrontal cortex of individuals with schizophrenia. Mol Psychiatry 2013; 18: 206–214. 22869038 10.1038/mp.2012.110

[bib95] Bayer TA, Buslei R, Havas L, Falkai P. Evidence for activation of microglia in patients with psychiatric illnesses. Neurosci Lett 1999; 271: 126–128. 10477118 10.1016/s0304-3940(99)00545-5

[bib96] Radewicz K, Garey LJ, Gentleman SM, Reynolds R. Increase in HLA-DR immunoreactive microglia in frontal and temporal cortex of chronic schizophrenics. J Neuropathol Exp Neurol 2000; 59: 137–150. 10749103 10.1093/jnen/59.2.137

[bib97] Togo T, Akiyama H, Kondo H, Ikeda K, Kato M, Iseki E et al. Expression of CD40 in the brain of Alzheimer's disease and other neurological diseases. Brain Res 2000; 885: 117–121. 11121537 10.1016/s0006-8993(00)02984-x

[bib98] Steiner J, Mawrin C, Ziegeler A, Bielau H, Ullrich O, Bernstein HG et al. Distribution of HLA-DR-positive microglia in schizophrenia reflects impaired cerebral lateralization. Acta Neuropathol 2006; 112: 305–316. 16783554 10.1007/s00401-006-0090-8

[bib99] Steiner J, Bernstein HG, Bielau H, Farkas N, Winter J, Dobrowolny H et al. S100B-immunopositive glia is elevated in paranoid as compared to residual schizophrenia: A morphometric study. J Psychiatr Res 2008; 42: 868–876. 18001771 10.1016/j.jpsychires.2007.10.001

[bib100] Busse S, Busse M, Schiltz K, Bielau H, Gos T, Brisch R et al. Different distribution patterns of lymphocytes and microglia in the hippocampus of patients with residual versus paranoid schizophrenia: Further evidence for disease course-related immune alterations? Brain Behav Immun 2012; 26: 1273–1279. 22917959 10.1016/j.bbi.2012.08.005

[bib101] Ji B, Maeda J, Sawada M, Ono M, Okauchi T, Inaji M et al. Imaging of peripheral benzodiazepine receptor expression as biomarkers of detrimental versus beneficial glial responses in Mouse Models of Alzheimer's and other CNS pathologies. J Neurosci 2008; 28: 12255–12267. 19020019 10.1523/JNEUROSCI.2312-08.2008 PMC2755188

[bib102] Cosenza-Nashat M, Zhao ML, Suh HS, Morgan J, Natividad R, Morgello S et al. Expression of the translocator protein of 18 kDa by microglia, macrophages and astrocytes based on immunohistochemical localization in abnormal human brain. Neuropathol Appl Neurobiol 2009; 35: 306–328. 19077109 10.1111/j.1365-2990.2008.01006.x PMC2693902

[bib103] Lavisse S, Guillermier M, Herard AS, Petit F, Delahaye M, Van Camp N et al. Reactive astrocytes overexpress TSPO and are detected by TSPO positron emission tomography imaging. J Neurosci 2012; 32: 10809–10818. 22875916 10.1523/JNEUROSCI.1487-12.2012 PMC6621018

[bib104] van Berckel BN, Bossong MG, Boellaard R, Kloet R, Schuitemaker A, Caspers E et al. Microglia activation in recent-onset Schizophrenia: a Quantitative (R)-[11C]PK11195 Positron Emission Tomography Study. Biol Psychiatry 2008; 64: 820–822. 18534557 10.1016/j.biopsych.2008.04.025

[bib105] Doorduin J, de Vries EFJ, Willemsen ATM, de Groot JC, Dierckx RA, Klein HC. Neuroinflammation in Schizophrenia-Related Psychosis: A PET Study. J Nucl Med 2009; 50: 1801–1807. 19837763 10.2967/jnumed.109.066647

[bib106] Takano A, Arakawa R, Ito H, Tateno A, Takahashi H, Matsumoto R et al. Peripheral benzodiazepine receptors in patients with chronic schizophrenia: a PET study with [11C]DAA1106. Int J Neuropsychopharmacol 2010; 13: 943–950. 20350336 10.1017/S1461145710000313

[bib107] Kenk M, Selvanathan T, Rao N, Suridjan I, Rusjan P, Remington G et al. Imaging neuroinflammation in gray and white matter in schizophrenia: An in-vivo PET study with [18F]-FEPPA. Schizophr Bull 2015; 41: 85–93. 25385788 10.1093/schbul/sbu157 PMC4266311

[bib108] Coughlin JM, Wang Y, Ambinder EB, Ward RE, Minn I, Vranesic M et al. *In vivo* markers of inflammatory response in recent-onset schizophrenia: a combined study using [11C]DPA-713 PET and analysis of CSF and plasma. Transl Psychiatry 2016; 6: e777. 27070405 10.1038/tp.2016.40 PMC4872398

[bib109] Van Der Doef TF, de Witte LD, Sutterland AL, Jobse E, Yaqub M, Boellaard R et al. *In vivo*(R) - [ 11 C ] PK11195 PET imaging of 18kDa translocator protein in recent onset psychosis. Npj Schizophr 2016; 2: 16031. 27602389 10.1038/npjschz.2016.31 PMC5007116

[bib110] Hafizi S, Tseng H-H, Rao N, Selvanathan T, Kenk M, Bazinet RP et al. Imaging microglial activation in untreated first-episode psychosis: A PET Study With [ ^18^ F]FEPPA. Am J Psychiatry 2016. 10.1176/appi.ajp.2016.16020171 PMC5342628 27609240

[bib111] Turkheimer FE, Rizzo G, Bloomfield PS, Howes O, Zanotti-Fregonara P, Bertoldo A et al. The methodology of TSPO imaging with positron emission tomography. Biochem Soc Trans 2015; 43: 586–592. 26551697 10.1042/BST20150058 PMC4613512

[bib112] Zhu F, Zheng Y, Ding YQ, Liu Y, Zhang X, Wu R et al. Minocycline and risperidone prevent microglia activation and rescue behavioral deficits induced by neonatal intrahippocampal injection of lipopolysaccharide in rats. PLoS One 2014; 9: e93966. 24705495 10.1371/journal.pone.0093966 PMC3976322

[bib113] Kato T, Mizoguchi Y, Monji A, Horikawa H, Suzuki SO, Seki Y et al. Inhibitory effects of aripiprazole on interferon-??-induced microglial activation via intracellular Ca2+ regulation *in vitro*. J Neurochem 2008; 106: 815–825. 18429930 10.1111/j.1471-4159.2008.05435.x

[bib114] Cotel M-C, Lenartowicz EM, Natesan S, Modo MM, Cooper JD, Williams SCR et al. Microglial activation in the rat brain following chronic antipsychotic treatment at clinically relevant doses. Eur Neuropsychopharmacol J Eur Coll Neuropsychopharmacol 2015; 25: 2098–2107. 10.1016/j.euroneuro.2015.08.004 26321204

[bib115] Bloomfield PS, Selvaraj S, Veronese M, Rizzo G, Bertoldo A, Owen DR et al. Microglial activity in people at ultra high risk of psychosis and in Schizophrenia: An [ 11 C]PBR28 PET Brain Imaging Study. Am J Psychiatry 2015. 10.1176/appi.ajp.2015.14101358 PMC4821370 26472628

[bib116] Holmes SE, Hinz R, Drake RJ, Gregory CJ, Conen S, Matthews JC et al. *In vivo* imaging of brain microglial activity in antipsychotic-free and medicated schizophrenia: a [11C](R)-PK11195 positron emission tomography study. Mol Psychiatry 2016; 21: 1672–1679. 27698434 10.1038/mp.2016.180

[bib117] Sandiego CM, Gallezot J-D, Pittman B, Nabulsi N, Lim K, Lin S-F et al. Imaging robust microglial activation after lipopolysaccharide administration in humans with PET. Proc Natl Acad Sci USA 2015; 112: 12468–12473. 26385967 10.1073/pnas.1511003112 PMC4603509

[bib118] Mondelli V, Cattaneo A, Belvederi Murri M, Di Forti M, Handley R, Hepgul N et al. Stress and inflammation reduce brain-derived neurotrophic factor expression in first-episode psychosis: a pathway to smaller hippocampal volume. J Clin Psychiatry 2011; 72: 1677–1684. 21672499 10.4088/JCP.10m06745 PMC4082665

[bib119] Goldsmith DR, Rapaport MH, Miller BJ. A meta-analysis of blood cytokine network alterations in psychiatric patients: comparisons between schizophrenia, bipolar disorder and depression. Mol Psychiatry 2016; 21: 1696–1709. 26903267 10.1038/mp.2016.3 PMC6056174

[bib120] Upthegrove R, Manzanares-Teson N, Barnes NM. Cytokine function in medication-naive first episode psychosis: A systematic review and meta-analysis. Schizophr Res 2014; 155: 101–108. 24704219 10.1016/j.schres.2014.03.005

[bib121] Yelmo-Cruz S, Morera-Fumero AL, Abreu-González P. S100B and schizophrenia. Psychiatry Clin Neurosci 2013; 67: 67–75. 23438158 10.1111/pcn.12024

[bib122] Baumeister D, Akhtar R, Ciufolini S, Pariante CM, Mondelli V. Childhood trauma and adulthood inflammation: a meta-analysis of peripheral C-reactive protein, interleukin-6 and tumour necrosis factor-α. Mol Psychiatry 2015; 21: 642–649. 26033244 10.1038/mp.2015.67 PMC4564950

[bib123] Chan MK, Krebs M-O, Cox D, Guest PC, Yolken RH, Rahmoune H et al. Development of a blood-based molecular biomarker test for identification of schizophrenia before disease onset. Transl Psychiatry 2015; 5: e601. 26171982 10.1038/tp.2015.91 PMC5068725

[bib124] Cannon TD, Chung Y, He G, Sun D, Jacobson A, Van Erp TGM et al. Progressive reduction in cortical thickness as psychosis develops: A multisite longitudinal neuroimaging study of youth at elevated clinical risk. Biol Psychiatry 2015; 77: 147–157. 25034946 10.1016/j.biopsych.2014.05.023 PMC4264996

[bib125] Najjar S, Pearlman DM. Neuroinfl ammation and white matter pathology in schizophrenia: systematic review. Schizophr Res 2015; 161: 102–112. 24948485 10.1016/j.schres.2014.04.041

[bib126] Fillman SG, Weickert TW, Lenroot RK, Catts S V, Bruggemann JM, Catts VS et al. Elevated peripheral cytokines characterize a subgroup of people with schizophrenia displaying poor verbal fluency and reduced Broca's area volume. Mol Psychiatry 2015; 21: 1–9. 10.1038/mp.2015.90 PMC4960447 26194183

[bib127] Ripke S, Neale BM, Corvin A, Walters JTR, Farh K-H, Holmans P A et al. Biological insights from 108 schizophrenia-associated genetic loci. Nature 2014; 511: 421–427. 25056061 10.1038/nature13595 PMC4112379

[bib128] Sekar A, Bialas AR, de Rivera H, Davis A, Hammond TR, Kamitaki N et al. Schizophrenia risk from complex variation of complement component 4. Nature 2016; 530: 177–183. 26814963 10.1038/nature16549 PMC4752392

[bib129] Stevens B, Allen NJ, Vazquez LE, Howell GR, Christopherson KS, Nouri N et al. The classical complement cascade mediates CNS synapse elimination. Cell 2007; 131: 1164–1178. 18083105 10.1016/j.cell.2007.10.036

[bib130] Fusar-Poli P, Radua J, McGuire P, Borgwardt S. Neuroanatomical maps of psychosis onset: Voxel-wise meta-analysis of antipsychotic-naive vbm studies. Schizophr Bull 2012; 38: 1297–1307. 22080494 10.1093/schbul/sbr134 PMC3494061

[bib131] Howes ODO, Kambeitz J, Stahl D, Slifstein M, Abi-Dargham A, Kapur S et al. The nature of dopamine dysfunction in schizophrenia and what this means for treatment. Arch Gen Psychiatry 2012; 69: 776–786. 22474070 10.1001/archgenpsychiatry.2012.169 PMC3730746

[bib132] Shepherd AM, Laurens KR, Matheson SL, Carr VJ, Green MJ. Systematic meta-review and quality assessment of the structural brain alterations in schizophrenia. Neurosci Biobehav Rev 2012; 36: 1342–1356. 22244985 10.1016/j.neubiorev.2011.12.015

[bib133] Sui J, Pearlson GD, Du Y, Yu Q, Jones TR, Chen J et al. In search of multimodal neuroimaging biomarkers of cognitive deficits in Schizophrenia. Biol Psychiatry 2015; 78: 794–804. 25847180 10.1016/j.biopsych.2015.02.017 PMC4547923

[bib134] Nestor PG, Kubicki M, Nakamura M, Niznikiewicz M, Levitt JJ, Shenton ME et al. Neuropsychological variability, symptoms, and brain imaging in chronic schizophrenia. Brain Imaging Behav 2013; 7: 68–76. 23011383 10.1007/s11682-012-9193-0

[bib135] Boksa P. Abnormal synaptic pruning in schizophrenia: Urban myth or reality? J Psychiatry Neurosci 2012; 37: 75–77. 22339991 10.1503/jpn.120007 PMC3297065

[bib136] Kim IH, Rossi M A, Aryal DK, Racz B, Kim N, Uezu A et al. Spine pruning drives antipsychotic-sensitive locomotion via circuit control of striatal dopamine. Nat Neurosci 2015; 18: 883–891. 25938885 10.1038/nn.4015 PMC4459733

[bib137] Grace AA. Dopamine system dysregulation by the hippocampus: Implications for the pathophysiology and treatment of schizophrenia. Neuropharmacology 2012; 62: 1342–1348. 21621548 10.1016/j.neuropharm.2011.05.011 PMC3179528

[bib138] Peciña M, Mickey BJ, Love T, Wang H, Langenecker SA, Hodgkinson C et al. DRD2 polymorphisms modulate reward and emotion processing, dopamine neurotransmission and openness to experience. Cortex 2013; 49: 877–890. 22424959 10.1016/j.cortex.2012.01.010 PMC3381848

[bib139] Niwa M, Jaaro-Peled H, Tankou S, Seshadri S, Hikida T, Matsumoto Y et al. Adolescent stress induced epigenetic control of Dopaminergic neurons via Glucocorticoids. Science 2013; 339: 335–340. 23329051 10.1126/science.1226931 PMC3617477

[bib140] Brake WG, Sullivan RM, Gratton A. Perinatal distress leads to lateralized medial prefrontal cortical dopamine hypofunction in adult rats. J Neurosci 2000; 20: 5538–5543. 10884337 10.1523/JNEUROSCI.20-14-05538.2000 PMC6772312

[bib141] Ribeiro BMM, do Carmo MRS, Freire RS, Rocha NFM, Borella VCM, de Menezes AT et al. Evidences for a progressive microglial activation and increase in iNOS expression in rats submitted to a neurodevelopmental model of schizophrenia: reversal by clozapine. Schizophr Res 2013; 151: 12–19. 24257517 10.1016/j.schres.2013.10.040

[bib142] Tynan RJ, Weidenhofer J, Hinwood M, Cairns MJ, Day TA, Walker FR. A comparative examination of the anti-inflammatory effects of SSRI and SNRI antidepressants on LPS stimulated microglia. Brain Behav Immun 2012; 26: 469–479. 22251606 10.1016/j.bbi.2011.12.011

[bib143] Xie N, Wang C, Lin Y, Li H, Chen L, Zhang T et al. The role of p38 MAPK in valproic acid induced microglia apoptosis. Neurosci Lett 2010; 482: 51–56. 20621161 10.1016/j.neulet.2010.07.004

[bib144] Inglese M, Petracca M. Therapeutic strategies in multiple sclerosis: a focus on neuroprotection and repair and relevance to schizophrenia. Schizophr Res 2015; 161: 94–101. 24893901 10.1016/j.schres.2014.04.040

[bib145] Meyer U, Schwarz MJ, Müller N. Inflammatory processes in schizophrenia: A promising neuroimmunological target for the treatment of negative/cognitive symptoms and beyond. Pharmacol Ther 2011; 132: 96–110. 21704074 10.1016/j.pharmthera.2011.06.003

[bib146] Wang B, Navath RS, Romero R, Kannan S, Kannan R. Anti-inflammatory and anti-oxidant activity of anionic dendrimer-N-acetyl cysteine conjugates in activated microglial cells. Int J Pharm 2009; 377: 159–168. 19463931 10.1016/j.ijpharm.2009.04.050 PMC3917717

[bib147] Vinet J, van Weering HR, Heinrich A, Kälin RE, Wegner A, Brouwer N et al. Neuroprotective function for ramified microglia in hippocampal excitotoxicity. J Neuroinflammation 2012; 9: 27. 22293457 10.1186/1742-2094-9-27 PMC3292937

[bib148] Abel KM, Drake R, Goldstein J. Sex differences in schizophrenia. Int J Soc Psychiatry 2010; 22: 417–428. 10.3109/09540261.2010.515205 21047156

[bib149] Arevalo MA, Santos-Galindo M, Bellini MJ, Azcoitia I, Garcia-Segura LM. Actions of estrogens on glial cells: Implications for neuroprotection. Biochim Biophys Acta - Gen Subj 2010; 1800: 1106–1112. 10.1016/j.bbagen.2009.10.002 19818384

[bib150] Cutando L, Busquets-Garcia A, Puighermanal E, Gomis-González M, Delgado-García JM, Gruart A et al. Microglial activation underlies cerebellar deficits produced by repeated cannabis exposure. J Clin Invest 2013; 123: 2816–2831. 23934130 10.1172/JCI67569 PMC3696568

[bib151] Di Forti M, Marconi A, Carra E, Fraietta S, Trotta A, Bonomo M et al. Proportion of patients in south London with first-episode psychosis attributable to use of high potency cannabis: a case-control study. Lancet Psychiatry 2015; 2: 233–238. 26359901 10.1016/S2215-0366(14)00117-5

[bib152] Catts VS, Fung SJ, Long LE, Joshi D, Vercammen A, Allen KM et al. Rethinking schizophrenia in the context of normal neurodevelopment. Front Cell Neurosci 2013; 7: 60. 23720610 10.3389/fncel.2013.00060 PMC3654207

[bib153] Howes O, Mccutcheon R, Stone J. Glutamate and dopamine in schizophrenia: An update for the 21 st century. J Psychopharmacol 2015; 29: 97–115. 25586400 10.1177/0269881114563634 PMC4902122

[bib154] Mondelli V, Ciufolini S, Murri MB, Bonaccorso S, Di Forti M, Giordano A et al. Cortisol and inflammatory biomarkers predict poor treatment response in first episode psychosis. Schizophr Bull 2015; 41: 1162–1170. 25829375 10.1093/schbul/sbv028 PMC4535637

