# Supplementary material for: Inflammation and the neural diathesis-stress hypothesis of schizophrenia: a reconceptualization
Source: Transl Psychiatry. 2017 Feb 7;7(2):e1024–. doi: 10.1038/tp.2016.278 (PMC5438023; doi:10.1038/tp.2016.278)
Supplement: Supplementary Table 1 [file tp2016278x1.docx]

| Study | Population (age) | Technique | Findings |
| --- | --- | --- | --- |
| Kurumaji et al 1997(1) | 13 Scz (60.1)  10 HC (66.7) | [3H] PK 11195 – ligand for peripheral type benzodiazepine receptor | Decreased binding in Scz in superior parietal cortex, primary visual area and putamen |
| Arnold et al. 1998 (2) | 23 Scz (79.8)  14 HC (75.3) | Ab for CD 68 | No significant differences between Scz and HC |
| Bayer et al. 1999 (3) | 14 Scz (64.2)  13 HC (57.5) | Ab for HLA-DR (TAL.1B5) | 3 Scz, 1 affective and 0 HC exhibited activated microglia |
| Falke et al. 2000 (4) | 11 Scz (80.5)  11 HC (77.6) | Ab for CD 68 | No significant differences between Scz and HC |
| Radewicz et al. 2000 (5) | 12 Scz (age 79.5)  12 HC (age 70.1) | Ab for HLA-DR (LN3) | Scz show increased activated microglia in the DLPFC, STG and ACG |
| Togo et al. 2000 (6) | 4 Scz  2 HC | Ab for HLA-DR (LN3)  Ab for CD40 (LOB7/6) | Unclear if any differences between Scz and HC |
| Wierzba-Bobrowicz et al. 2004 (7) | 12 Scz (59.1)  7 HC (55.6) | Ab for HLA-DP/DQ/DR (CR3/43) | Microglia in Scz qualitatively show more ‘degenerative changes’ |
| Wierzba-Bobrowicz et al. 2005 (8) | 9 Scz (55.7)  6 HC (56.3) | Ab for HLA-DP/DQ/DR (CR3/43) | Scz show greater number of activated microglia with greater proportion of ‘damaged processes’ |
| Foster et al. 2006 (9) | 15 Scz (44)  15 HC (48) | Ab for CD68 and calprotectin | No difference in CD68 levels, but calprotectin increased in Scz |
| Steiner et al. 2006 (10) | 16 Scz (54.5)  16 HC (57.7) | Ab for HLA-DR (TAL.1B5) | No group difference in in microglial density. Highly raised microglial numbers in pts who completed suicide during acute psychosis |
| Steiner et al 2008 (11) | 16Scz (12 subjects from Steiner et al 2006) (53) 10 HC (54) | Ab for HLA-DR (TAL.1B5) | No effect of diagnosis but increased microglial density in DLPFC, ACC and mediodorsal thalamus of patients who completed suicide. |
| Connor et al 2009 (12) | 22 Scz (68)  45 HC (70) | Antibody for Iba1 | No differences in Iba 1 reactivity between groups |
| Uranova et al. 2011(13) | 40 Scz (58.7)  40 HC (55.5) | Electron microscopy | Microglia involved in phagocytosis of myelin sheets in Scz but not in HC |
| Busse et al. 2012 (14) | 17 Scz (52)  11 HC (56) | Ab for HLA-DR (TAL.1B5) | Increased microglial density only in paranoid as opposed to residual subtype |
| Fillman et al. 2013 (15) | 37 Scz (51.3)  37 HC (51.1) | Ab for HLA-DP/DQ/DR  Microglial mRNA markers | Increased microglial density in Scz DLPFC |
| Rao et al. 2013 (16) | 10 Scz (59)  10 HC (49) | Gene expression of CD11b & Ab for HLD-A | Increased markers in Scz |
| Gos et al. 2014 (17) | 13 Scz (51)  12 HC (49) | Ab for HLA-DR and quinolinic acid | Reduce quinolinic acid binding in hippocampus. |
| Hercher et al. 2014 (18) | 20 Scz (44.7)  20 HC (45.3) | Ab for Iba1 | No differences between groups in terms of microglial density.  Morphological assessment found activated microglia in 3 Scz but none in the other groups. |
| Durrenberger et al. 2015 (19) | 10 Scz (66)  10 HC (61) | Gene Expression of HLA-DR | Reduced HLA-DR expression in schizophrenia |

**Supplementary Table 1: Post mortem studies investigating microglia density and activation in individuals with psychotic disorders**

Ab – antibody; ACG – Anterior cingulate gyrus; CD68 – cluster of differentiation 68; DLPFC – dorsolateral prefrontal corte;. HC – Healthy Control; HLA – human leukocyte antigen; Iba1 - ionized calcium-binding adapter molecule 1 Scz – Schizophrenia; STG – superior temporal gyrus

1. Kurumaji A, Wakai T, Toru M. Decreases in peripheral-type benzodiazepine receptors in postmortem brains of chronic schizophrenics. J Neural Transm. 1997;104(11–12):1361–70.

2. Arnold SE, Trojanowski JQ, Gur RE, Blackwell P, Han LY, Choi C. Absence of neurodegeneration and neural injury in the cerebral cortex in a sample of elderly patients with schizophrenia. Arch Gen Psychiatry. 1998;55(3):225–32.

3. Bayer TA, Buslei R, Havas L, Falkai P. Evidence for activation of microglia in patients with psychiatric illnesses. Neurosci Lett. 1999;271(2):126–8.

4. Falke E, Han LY, Arnold SE. Absence of neurodegeneration in the thalamus and caudate of elderly patients with schizophrenia. Psychiatry Res. 2000;93(2):103–10.

5. Radewicz K, Garey LJ, Gentleman SM, Reynolds R. Increase in HLA-DR immunoreactive microglia in frontal and temporal cortex of chronic schizophrenics. J Neuropathol Exp Neurol. 2000;59(2):137–50.

6. Togo T, Akiyama H, Kondo H, Ikeda K, Kato M, Iseki E, et al. Expression of CD40 in the brain of Alzheimer’s disease and other neurological diseases. Brain Res. 2000;885(1):117–21.

7. Wierzba-Bobrowicz T, Lewandowska E, Kosno-Kruszewska E, Lechowicz W, Pasennik E, Schmidt-Sidor B. Degeneration of microglial cells in frontal and temporal lobes of chronic schizophrenics. Folia Neuropathol. 2004 Jan;42(3):157–65.

8. Wierzba-Bobrowicz T, Lewandowska E, Lechowicz W, Stepień T, Pasennik E. Quantitative analysis of activated microglia, ramified and damage of processes in the frontal and temporal lobes of chronic schizophrenics. Folia Neuropathol. 2005 Jan;43(2):81–9.

9. Foster R, Kandanearatchi A, Beasley C, Williams B, Khan N, Fagerhol MK, et al. Calprotectin in microglia from frontal cortex is up-regulated in schizophrenia: Evidence for an inflammatory process? Eur J Neurosci. 2006;24(12):3561–6.

10. Steiner J, Mawrin C, Ziegeler A, Bielau H, Ullrich O, Bernstein HG, et al. Distribution of HLA-DR-positive microglia in schizophrenia reflects impaired cerebral lateralization. Acta Neuropathol. 2006;112(3):305–16.

11. Steiner J, Bielau H, Brisch R, Danos P, Ullrich O, Mawrin C, et al. Immunological aspects in the neurobiology of suicide: Elevated microglial density in schizophrenia and depression is associated with suicide. J Psychiatr Res. 2008;42(2):151–7.

12. Connor CM, Guo Y, Akbarian S. Cingulate White Matter Neurons in Schizophrenia and Bipolar Disorder. Biol Psychiatry. 2009;66(5):486–93.

13. Uranova N a., Vikhreva O V., Rachmanova VI, Orlovskaya DD. Ultrastructural Alterations of Myelinated Fibers and Oligodendrocytes in the Prefrontal Cortex in Schizophrenia: A Postmortem Morphometric Study. Schizophr Res Treatment. 2011;2011:1–13.

14. Busse S, Busse M, Schiltz K, Bielau H, Gos T, Brisch R, et al. Different distribution patterns of lymphocytes and microglia in the hippocampus of patients with residual versus paranoid schizophrenia: Further evidence for disease course-related immune alterations? Brain Behav Immun. Elsevier Inc.; 2012;26(8):1273–9.

15. Fillman SG, Cloonan N, Catts VS, Miller LC, Wong J, McCrossin T, et al. Increased inflammatory markers identified in the dorsolateral prefrontal cortex of individuals with schizophrenia. Mol Psychiatry. Nature Publishing Group; 2013;18(2):206–14.

16. Rao JS, Kim HW, Harry GJ, Rapoport SI, Reese EA. Increased neuroinflammatory and arachidonic acid cascade markers, and reduced synaptic proteins, in the postmortem frontal cortex from schizophrenia patients. Schizophr Res. Elsevier B.V.; 2013;147(1):24–31.

17. Gos T, Myint AM, Schiltz K, Meyer-Lotz G, Dobrowolny H, Busse S, et al. Reduced microglial immunoreactivity for endogenous NMDA receptor agonist quinolinic acid in the hippocampus of schizophrenia patients. Brain Behav Immun. Elsevier Inc.; 2014;41(1):59–64.

18. Hercher C, Chopra V, Beasley CL. Evidence for morphological alterations in prefrontal white matter glia in schizophrenia and bipolar disorder. J Psychiatry Neurosci. 2014;39(6):376–85.

19. Durrenberger PF, Fernando FS, Kashefi SN, Bonnert TP, Seilhean D, Nait-Oumesmar B, et al. Common mechanisms in neurodegeneration and neuroinflammation: a BrainNet Europe gene expression microarray study. J Neural Transm. 2014;122(7):1055–68.
